# Heavy metals in locus ceruleus and motor neurons in motor neuron disease

**DOI:** 10.1186/2051-5960-1-81

**Published:** 2013-12-12

**Authors:** Roger Pamphlett, Stephen Kum Jew

**Affiliations:** 1 Department of Pathology, The Stacey Motor Neuron Disease Laboratory, Sydney Medical School, The University of Sydney, Sydney, Australia

**Keywords:** Motor neuron disease, Amyotrophic lateral sclerosis, Toxicant, Heavy metal, Neurotoxin, Locus ceruleus, Motor neurons, Autometallography, Mercury, Sporadic ALS, Familial ALS

## Abstract

**Background:**

The causes of sporadic amyotrophic lateral sclerosis (SALS) and other types of motor neuron disease (MND) remain largely unknown. Heavy metals have long been implicated in MND, and it has recently been shown that inorganic mercury selectively enters human locus ceruleus (LC) and motor neurons. We therefore used silver nitrate autometallography (AMG) to look for AMG-stainable heavy metals (inorganic mercury and bismuth) in LC and motor neurons of 24 patients with MND (18 with SALS and 6 with familial MND) and in the LC of 24 controls.

**Results:**

Heavy metals in neurons were found in significantly more MND patients than in controls when comparing: (1) the presence of any versus no heavy metal-containing LC neurons (MND 88%, controls 42%), (2) the median percentage of heavy metal-containing LC neurons (MND 9.5%, control 0.0%), and (3) numbers of individuals with heavy metal-containing LC neurons in the upper half of the percentage range (MND 75%, controls 25%). In MND patients, 67% of remaining spinal motor neurons contained heavy metals; smaller percentages were found in hypoglossal, nucleus ambiguus and oculomotor neurons, but none in cortical motor neurons. The majority of MND patients had heavy metals in both LC and spinal motor neurons. No glia or other neurons, including neuromelanin-containing neurons of the substantia nigra, contained stainable heavy metals.

**Conclusions:**

Uptake of heavy metals by LC and lower motor neurons appears to be fairly common in humans, though heavy metal staining in the LC, most likely due to inorganic mercury, was seen significantly more often in MND patients than in controls. The LC innervates many cell types that are affected in MND, and it is possible that MND is triggered by toxicant-induced interactions between LC and motor neurons.

## Background

The causes of most cases of amyotrophic lateral sclerosis (ALS), the most common form of motor neuron disease (MND), as well as the other subtypes of MND, are still unknown [1]. So far only a modest proportion of sporadic MND patients have been found to harbour causative genetic mutations, and attention has again turned to the possibility that in some patients the disease may be caused by an environmental toxicant that affects motor neurons preferentially. The recent finding that one metal toxicant, inorganic mercury, enters the human locus ceruleus (LC) and motor neurons selectively after mercury self-injection suggests this pathway could be used by toxicants to enter and damage motor neurons [2].

The LC has many interactions with motor neurons so it is possible that some combination of toxicant uptake by the LC and motor neurons might underlie MND. A previous study of ours using the histochemical technique of autometallographic silver amplification (AMG) found heavy metals in spinal motor neurons in patients with MND and controls, but the LC was not examined in that study [3]. We therefore used AMG to examine heavy metals in both LC and spinal motor neurons, using tissue from previously-reported as well as from additional MND patients in whom paraffin blocks containing the LC were available. Updated histological criteria for heavy metal staining and new quantitative procedures for the presence of heavy metals in neurons were employed. The CNS was also sampled widely to see if heavy metal staining was specific for LC and motor neurons.

## Methods

### Nomenclature

(1) The term “toxicant” is used to describe a poison that is put into the environment by human activity, in contrast to a toxin, which is a poison produced naturally by an organism; the term toxicant does not necessarily imply damage to cells, since the toxicant may be at too low a concentration to cause cell injury. (2) The term “heavy metal” in this study refers to the two metals that can be stained using the routine AMG process, inorganic mercury and bismuth. Although it seems most likely that the metal demonstrated by AMG in this study is inorganic mercury (since exposure to bismuth is uncommon), we use the term metal toxicant or heavy metal instead of inorganic mercury since we cannot definitively identify the metal concerned using paraffin sections. (3) Unless otherwise stated, the term “locus ceruleus” (LC) refers to the locus ceruleus nucleus in the pons, to prevent confusion with the nucleus of an individual LC neuron.

### Cases and controls

#### Cases

Cases were 24 patients (13 male, 11 female, age range 38–84 years, mean age 61 years SD 13 years) with a clinical diagnosis of MND, made by a neurologist, where the diagnosis was confirmed pathologically on *post mortem* examination of brain and spinal cord tissue performed between 1988 and 1999 (Table 1). MND cases were restricted to those in whom available formalin-fixed paraffin-embedded blocks contained: (1) Neuromelanin-containing neurons of the locus ceruleus in the rostral pons. (2) Anterior horn motor neuron cell bodies in the spinal cord (lumbar and/or cervical). (3) Hypoglossal motor neurons in the medulla oblongata. In MND patients tissue blocks were also available from the frontal motor cortex, which had been sampled horizontally to include the maximum number of corticomotor neurons [4]), occipital cortex, midbrain, hippocampus, caudate-putamen, and cerebellum.

**Table 1:** Details of heavy metal staining in LC and motor neurons of MND patients and controls

**Group**	**Diagnosis**	**Age**	**Gender**	**LC no. Pos**	**LC no. Neg**	**LC% Pos**	**LC% Pos Category**	**SMN AMG grade**	**12n AMG grade**	**NAm AMG grade**	**3n/4n AMG grade**
MND	SALS	71	Female	0	68	0	Low	-	-	-	4n-
	SALS	62	Female	1	72	1	Low	-	-	-	4n+
	SALS	66	Female	2	90	2	Low	+	+	-	4n-
	SALS	38	Male	4	81	5	High	-	-	-	NA
	SALS	77	Female	4	72	5	High	+	-	-	4n+
	SALS	81	Female	5	52	9	High	++	++	++	3n+
	SALS	59	Female	14	82	15	High	-	-	-	4n-
	SALS	46	Male	12	60	17	High	++	+	+	4n+
	SALS	67	Female	17	66	20	High	++	++	++	4n-
	SALS	45	Male	13	46	22	High	-	-	++	4n-
	SALS C9orf72	74	Male	15	48	24	High	-	-	-	4n-
	SALS	60	Female	32	79	29	High	+	+	-	3n-
	SALS	49	Male	27	57	32	High	+	+	++	NA
	SPMA C9orf72	60	Female	0	60	0	Low	+	+	-	4n-
	SPMA	58	Male	11	97	10	High	++	-	-	4n+
	SPMA	84	Male	21	64	25	High	+	-	-	3n-
	SPMA	74	Male	23	48	32	High	++	-	+	4n+
	SPMA	53	Male	56	14	80	High	+	+	-	4n-
	FMND	70	Male	21	75	22	High	-	-	-	4n-
	FMND C9orf72	59	Male	2	32	6	High	+	-	+	4n-
	FMND C9orf72	76	Female	5	79	6	High	+	+	-	4n-
	FMND SOD1	41	Male	0	50	0	Low	++	-	-	4n-
	FMND SOD1	47	Female	4	90	4	High	++	+	++	4n-
	FMND Kennedy	53	Male	2	101	2	Low	-	-	-	4n-
Control	Normal	86	Female	0	35	0	Low	+	+	+	NA
	Normal	55	Female	0	38	0	Low	-	-	-	NA
	Normal	77	Male	0	42	0	Low	-	NA	NA	NA
	Normal	24	Male	0	122	0	Low	-	NA	NA	NA
	Normal	82	Female	0	66	0	Low	NA	NA	NA	NA
	Normal	77	Female	0	29	0	Low	NA	NA	NA	NA
	Normal	44	Male	0	90	0	Low	NA	NA	NA	NA
	Normal	30	Female	0	82	0	Low	NA	NA	NA	NA
	Normal	75	Female	0	45	0	Low	NA	NA	NA	NA
	Normal	71	Male	1	74	1	Low	NA	NA	NA	NA
	Normal	47	Female	1	57	2	Low	NA	NA	NA	NA
	Normal	77	Female	2	98	2	Low	NA	NA	NA	NA
	Normal	55	Male	3	99	3	Low	NA	NA	NA	NA
	Normal	26	Male	3	54	5	High	+	+	-	NA
	Normal	52	Female	6	75	7	High	NA	NA	NA	NA
	Normal	54	Male	12	65	16	High	NA	NA	NA	NA
	Normal	74	Male	9	35	20	High	-	-	-	4n+
	Alcohol	36	Male	0	58	0	Low	NA	NA	NA	NA
	Alcohol	49	Female	0	70	0	Low	NA	NA	NA	NA
	Alcohol	33	Male	20	57	26	High	NA	NA	NA	NA
	Multiple sclerosis	51	Female	17	69	20	High	NA	NA	NA	NA
	Parkinson dis.	83	Male	0	13	0	Low	+	NA	NA	3n++
	Parkinson dis.	72	Female	0	12	0	Low	NA	NA	NA	4n+
	Parkinson dis.	74	Male	0	11	0	Low	NA	NA	NA	4n-

3n: Oculomotor neurons, 4n: Trochlear neurons, 12n: Hypoglossal neurons, *NA*: Not available, *NAm*: Nucleus ambiguus neurons, *SMN*: Spinal motor neurons (see text for other abbreviations).

The results of neurological examinations were incomplete in some of the archival cases, though family histories of MND were available from all. During this period of tissue collection the only genetic tests available were for mutations in the gene for superoxide-dismutase 1 (SOD1) and for repeat expansions in the androgen receptor gene for Kennedy’s disease, and no frozen tissue was available for DNA extraction. We therefore immunostained spinal cord sections with TDP-43 and CD-68, and hippocampal and cerebellar sections with p62 and TDP-43 to enable classification as: (1) Sporadic or familial MND (FMND), based on family history. (2) Classic ALS, based on upper and lower motor neuron loss, TDP-43 inclusions in spinal motor neurons, and increased microglia in the lateral corticospinal tracts on CD-68 staining. (3) Sporadic progressive muscular atrophy (SPMA) variant of MND, based on lower motor neuron loss, TDP-43 inclusions in remaining spinal motor neurons, and no increase in microglia in the lateral corticospinal tracts on CD-68 staining [5]. Familial MND was further classified into those with: (1) Mutations in *SOD1* or the androgen receptor gene. (2) Probable *C9orf72* mutations, based on the characteristic p62 neuronal inclusions in the hippocampus or cerebellum [6]. (3) Unknown mutations, for patients who did not fit into the previous two categories.

#### Controls

Controls were 24 individuals (12 male, 12 female, age range 24-86 years, mean age 59 years SD 20 years) in whom paraffin blocks containing the locus ceruleus were available, from the same sources and the same time period as the MND blocks (Table 1). Controls consisted of: (1) Seventeen individuals who died from non-neurological conditions where no brain pathology was found. (2) Three individuals who met the DSMIV criteria for alcohol abuse, but who had no brain pathology. (3) Three individuals with Parkinson’s disease. (4) One individual with multiple sclerosis. In addition to the control LC blocks, 6 controls had midbrain blocks containing the substantia nigra, 6 had spinal cord blocks, and 4 had medulla oblongata blocks.

The project was approved by the Human Ethics Committee of Sydney South West Area Health Service.

### Autometallography

7 μm paraffin sections were stained for metal toxicants using silver nitrate autometallography (AMG), a silver amplification method which under routine conditions stains the sulphides or selenides of mercury, silver, and bismuth [7,8]. Briefly, paraffin sections were placed in physical developer containing 50% gum arabic, citrate buffer, hydroquinone and silver nitrate at 26°C for 80 min in the dark, then washed in 5% sodium thiosulphate to remove unbound silver. Sections were counterstained with Harris hematoxylin and viewed under bright-field illumination. Because Harris haematoxylin contains a small amount of mercuric oxide, sections were also counterstained with mercury-free Improved Harris haematoxylin, but no difference in AMG staining was seen. Silver-coated metal deposits were seen as black-staining grains. In each staining run, a positive control section was included of mouse spinal cord motor neurons which contained mercury deposits after an intraperitoneal injection of 2 μg/g mercuric chloride [9]. Parallel sections of selected AMG-staining sections were pretreated for 2 h with 1% potassium cyanide to distinguish any silver deposits, which disappear after this treatment [10].

AMG staining of individual neurons was graded as: (1) Negative, when there were no or fewer than 10 AMG grains per neuron. (2) Light, when there were 10 or more AMG grains restricted to pigment-containing (either neuromelanin or lipofuscin) regions of the neuron. (3) Dense, when there were more than 10 AMG grains in non-pigmented regions of the neuron, or when the grains were too compact to judge whether any underlying neuronal pigment was present.

### Quantitation of locus ceruleus neurons containing AMG grains

To compare the percentage of AMG-stained LC neurons between MND patients and controls, a 10×10 grid, with right and lower exclusion margins, viewed at 400× magnification, was stepped sequentially through the LC. To be included for quantitation, an AMG-stained LC neuron was defined as any neuron (pigmented or non-pigmented) with a maximum diameter greater than 26 μm (the length of the side of one grid square) that contained 10 or more AMG grains. Neurons counted were those enclosed by an area bordered by the LC pigmented neurons. Large (greater than 26 μm in diameter) non-pigmented neurons made up about one-quarter of the neuronal count. The numbers of neurons counted varies between cases mostly because the area of the LC varies greatly throughout its length. A pilot study indicated that the percentage of AMG-stained LC neurons was similar between sides, so only one randomly-selected side was counted. The quantifier (RP) was blinded to the diagnosis by having the label on each slide obscured. One slide counted on 10 different occasions gave a coefficient of error of less than 5%.

The small number of spinal motor neurons in SMND patients did not allow for formal quantitation of AMG staining.

### Statistics

GraphPad Prism 5 was used to analyse contingency tables with Fisher’s exact test. Non-Gaussian continuous variable results were analysed with Mann-Whitney nonparametric tests. Double-sided p values were calculated with alpha <0.05.

## Results

### Heavy metal staining in the brain and brain stem

The only CNS cells to stain for heavy metals were neurons in the LC and motor neurons in the spinal cord and brain stem. Heavy metal staining was not removed by potassium cyanide pre-treatment, indicating the staining was not due to silver deposits [10]. This indicates that the standard AMG method used in this study demonstrated either mercury or bismuth in the cells [11,12]. A summary of heavy metal staining in each individual is shown in Table 1.

### Heavy metals in the locus ceruleus

The density of neurons in cross-sections of the LC of MND patients appeared similar to those of the normal controls, as far as could be judged visually on one (variable) level of the LC without formal quantitation. In the three Parkinson’s disease patients, however, LC neuronal density was reduced by about 80% when compared to normal control counts at the same horizontal level of the pons, leaving only a few scattered surviving neurons.

#### Heavy metals in LC neurons

**Figure 1:**
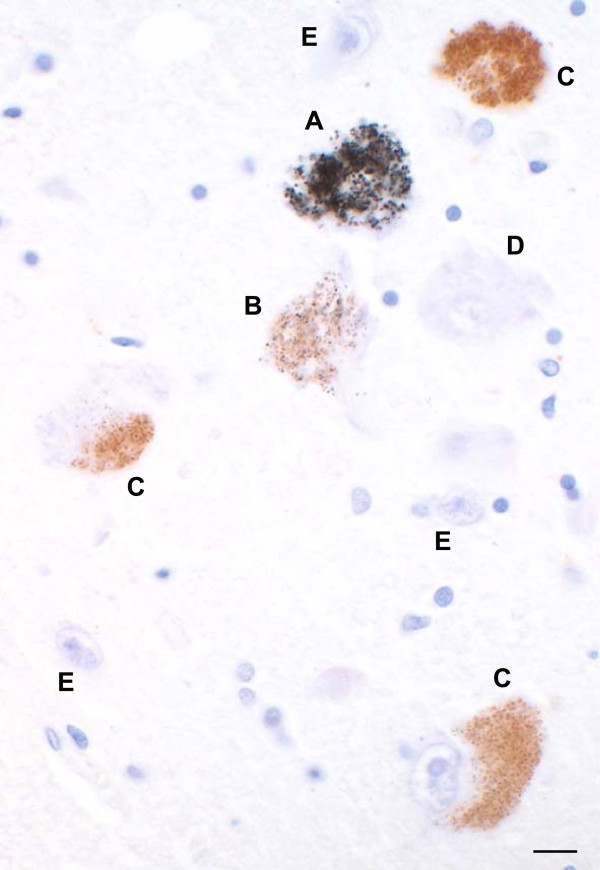
**Heavy metal staining in the LC of a MND patient.** **(A)** One LC neuron in this field stains densely with AMG, making it difficult to judge whether the neuromelanin is obscured or whether this is a non-pigmented neuron. **(B)** A pigmented neuron with light AMG staining, seen as more than 10 small black grains within the yellow-brown neuromelanin. **(C)** Three pigmented neurons with no quantifiable AMG grains (none in the two neurons on the right, and fewer than 10 grains in the neuron on the left). **(D)** A large non-pigmented neuron with no heavy metal staining. **(E)** Three small non-pigmented neurons with no heavy metal staining. No glial cells in the LC stain for heavy metals. Bar = 15 μm. AMG and hematoxylin.

Either no, light, or dense heavy metal staining could be seen in LC neurons (Figure 1). A mosaic of LC neurons containing no, light or dense heavy metal staining was usually seen. LC neurons with heavy metals mostly contained neuromelanin granules as well. In some neurons dense heavy metal staining made it difficult to judge whether or not the neuron contained any neuromelanin (Figure 1). No glial cells within the LC contained heavy metals.

#### Comparison between heavy metal staining in MND and control LC neurons

**Figure 2:**
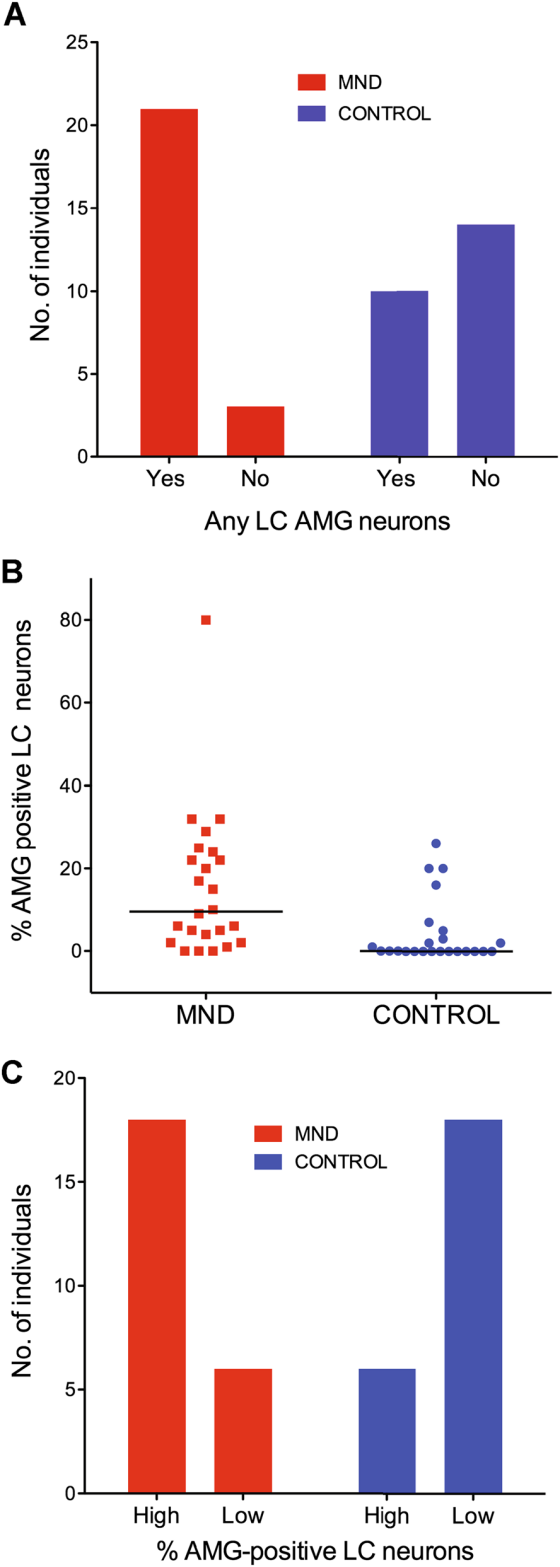
**Comparison of LC heavy metal staining between MND patients and controls. (A)** The number of MND patients having any heavy metal staining neurons in their LC was greater than in controls (chi square p = 0.002). **(B)** LC neurons containing heavy metal staining were present in both MND and control groups, but were significantly higher in the MND group (Mann–Whitney p = 0.0006). Bars = median values. **(C)** When the percentages of LC neurons containing heavy metals were divided into high (upper 50% of range) and low (lower 50% of range) categories, MND patients were predominantly in the high category, significantly different to the mirror-image result in controls (chi-square p = 0.0012).

Significantly more MND patients (88%) than controls (42%) had an LC that contained at least one neuron with heavy metal staining (chi square p = 0.002) (Figure 2A). In addition, MND patients had significantly greater percentages of LC neurons with heavy metals than controls (MND median 9.5%, control median 0.0%, Mann–Whitney p = 0.0006) (Figure 2B).

In both MND patients and controls, some LC contained very few heavy metal-stained neurons, which are likely to be insufficient in number to be of biological importance. Percentages of LC neurons containing heavy metals were therefore divided into upper and lower halves, based on a median value for the whole cohort of 4% stained neurons per individual. The low-percentage of AMG-stained neurons (low^%AMG^) category had 0% to 3% heavy metal-stained LC neurons per individual, and the high-percentage (high^%AMG^) category 4% or more heavy metal-stained LC neurons per individual. The LC of MND patients were significantly more likely to be in the high^%AMG^ category (75%) than those of controls (25%) (chi-square p = 0.0012) (Figure 2C).

#### Comparison of heavy metal-stained LC neurons in MND subtypes

**Figure 3:**
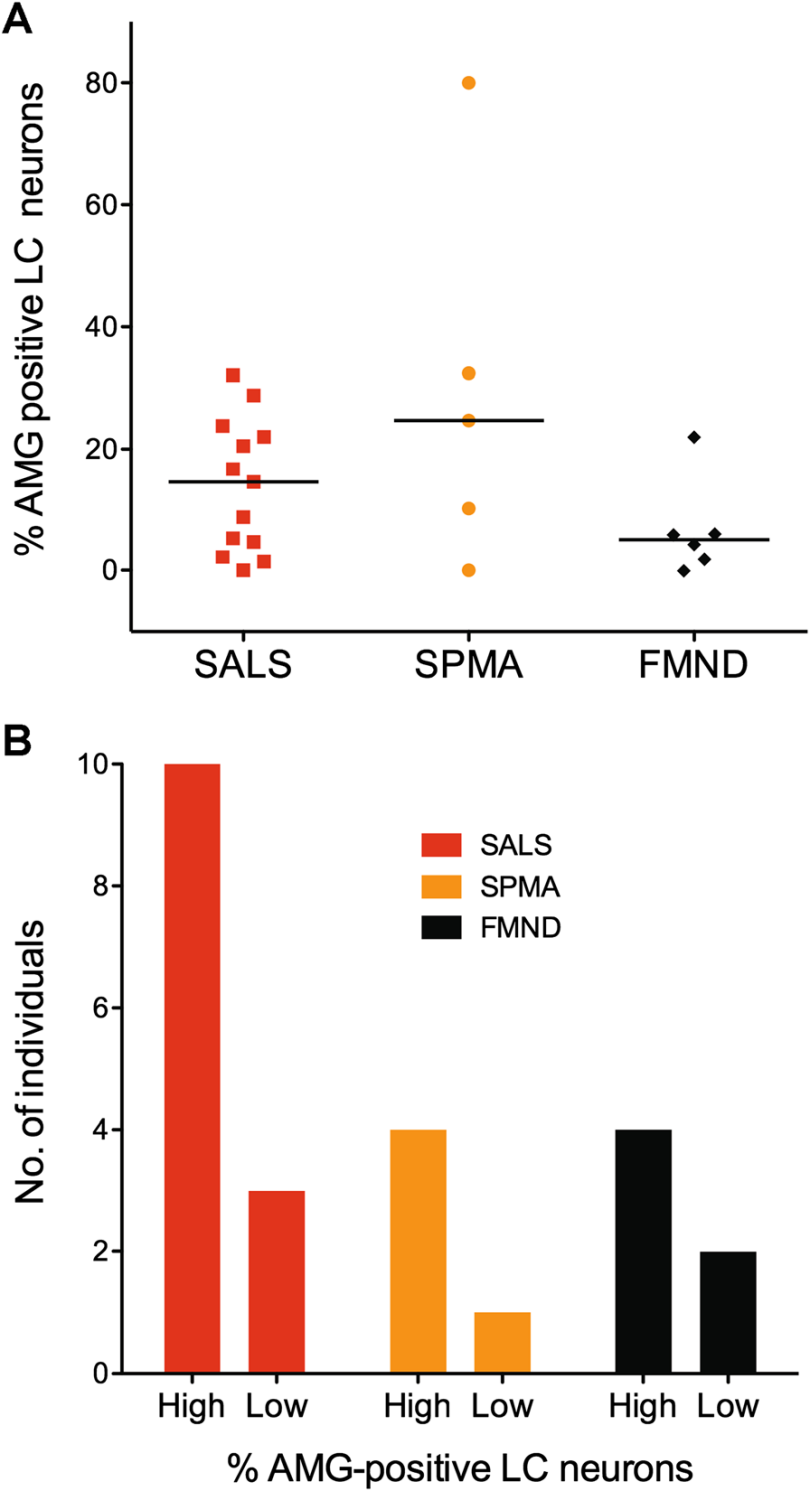
**Comparison of LC heavy metal staining in SALS, SPMA and FMND patients. (A)** The percentage of LC neurons containing heavy metals varied widely in all MND subgroups, particularly in the SMND group, but was not significantly different between groups. Bars = median values. **(B)** All MND subgroups had more high (upper 50% of range) than low (lower 50% of range) category LC neurons containing heavy metals, with no significant differences between groups.

The percentage of heavy metal-stained LC neurons per individual varied widely in all three MND subgroups, in particular in the sporadic progressive muscular atrophy (SPMA) subgroup (Figure 3A). Low^%AMG^ and high^%AMG^ categories were seen in similar numbers in all three subgroups (Figure 3B).

All except 1 of the 13 SALS patients had LC nuclei in the high^%AMG^ category. The MND patient with the highest percentage of heavy metal-stained LC neurons (80%) had SPMA.

Five of the 6 familial MND (FMND) patients had heavy metal-stained LC neurons, with 4 of these in the high^%AMG^ category.

#### Heavy metals in LC neurons: the effects of age and gender

To look for age effects on heavy metal staining in the LC, the cohort was divided into younger (24–59 years) and older (60–86 years) groups, based on a median age of 59.5 years. The group in the lower half of the age range had a slightly greater proportion of LC nuclei in the high^%AMG^ category (58%) compared to the older group (42%), but this did not reach statistical significance (chi-square p = 0.39). Heavy metals did not therefore appear to accumulate in the LC with age. The youngest individual to have AMG-stained LC neurons was a 26 year-old normal control individual, and the oldest an 84 year-old MND patient.

More males (61%) had LC in the high^%AMG^ category than females (39%), but this did not reach statistical significance (chi-square p = 0.25).

#### Heavy metals in the LC in sporadic and familial MND

Four out of 6 (67%) FMND patients, compared to 15 out of 18 (83%) sporadic MND patients, had LC in the high^%AMG^ category. Therefore the majority of patients in both familial and sporadic MND groups had heavy metals in the LC in the high^%AMG^ category (chi-square analysis not performed due to small numbers in the FMND group). No uniformity of LC AMG staining was present in *SOD1* mutant or *C9orf72* mutant MND patient groups, with both high^%AMG^ and low^%AMG^ categories within these groups.

### Heavy metals in motor neurons

#### Spinal motor neurons

**Figure 4:**
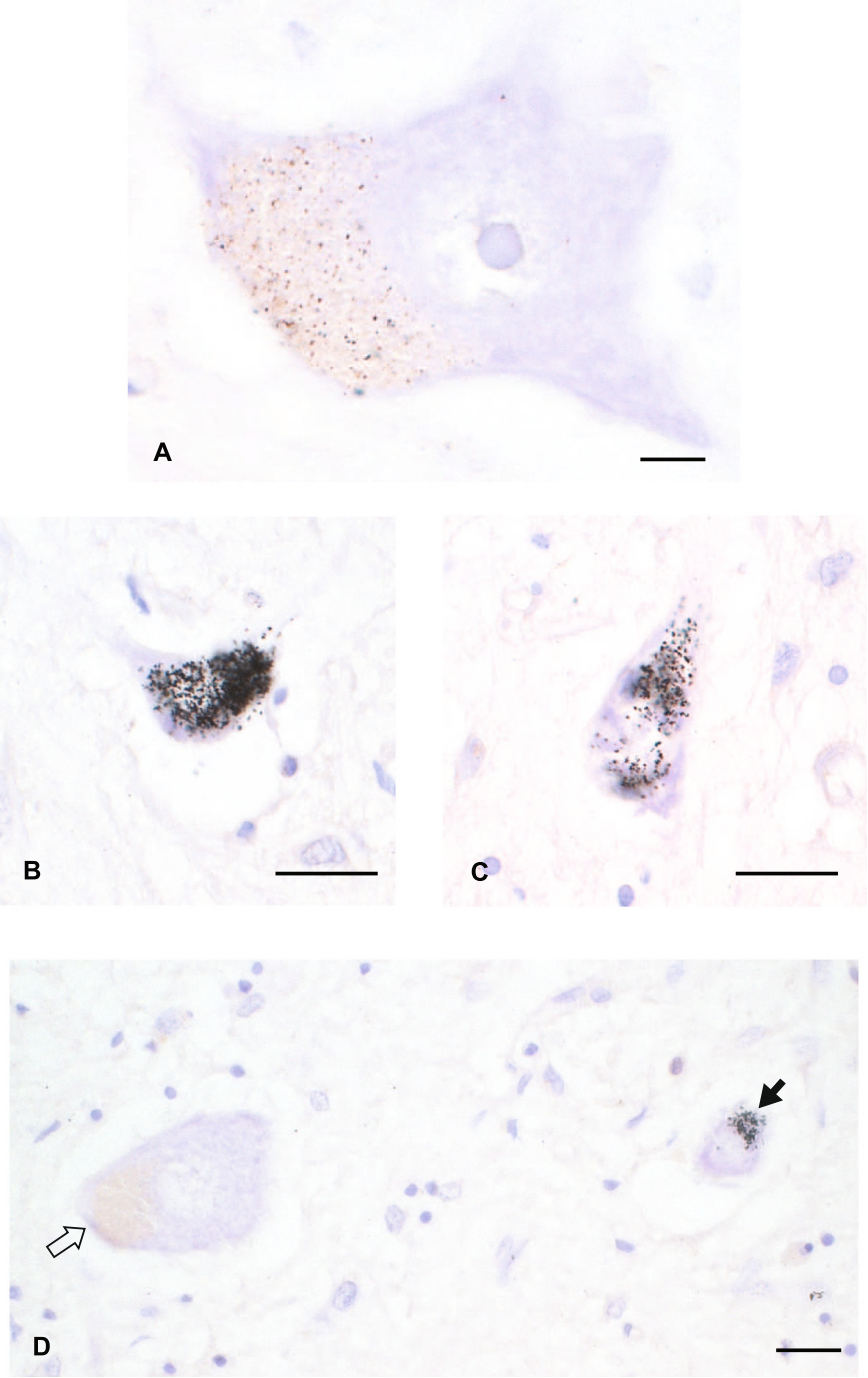
**Heavy metal staining in spinal motor neurons of MND patients (B, C and D from the same patient in Figure**1**). (A)** Light AMG staining can be seen within the lipofuscin of this normal-appearing spinal motor neuron, but none is present in the non-pigmented cytoplasm. Bar = 7 μm. **(B)** and **(C)** Two shrunken spinal motor neurons with dense AMG staining, which is either obscuring the lipofuscin, or is free in the cytoplasm. Bars = 20 μm. **(D)** A shrunken spinal motor neuron contains dense heavy metal staining (closed arrow), whereas an adjacent normal-sized neuron (open arrow) contains no heavy metal staining, despite containing lipofuscin. Bar = 15 μm. AMG and hematoxylin.

All MND patients had severe losses of anterior horn spinal motor neurons, with fewer than 10 surviving neurons in some patients. No formal quantitation of heavy metal-stained spinal motor neurons could therefore be undertaken. Sixteen (67%) of the 24 MND patients had heavy metals in their spinal motor neurons; in 9 of these metal staining was light (Figure 4A), and in 7 dense (Figure 4B, C). Dense AMG staining was seen particularly in smaller neurons, with adjacent normal-sized neurons often showing no or only light AMG staining (Figure 4D).

Heavy metals were seen in spinal motor neurons of all MND subgroups (SALS, SPMA and FMND). Three (54%) of the 7 controls had lightly AMG-stained spinal motor neurons.

#### Heavy metals in both LC and spinal motor neurons

Heavy metals were present in both LC and spinal motor neurons in 14 (58%) of the 24 MND patients. The majority of MND patients with LC AMG staining therefore also had spinal motor neuron AMG staining. Only 1 (17%) of the 6 control patients had both LC and spinal motor AMG staining.

#### Heavy metals in hypoglossal motor neurons

**Figure 5:**
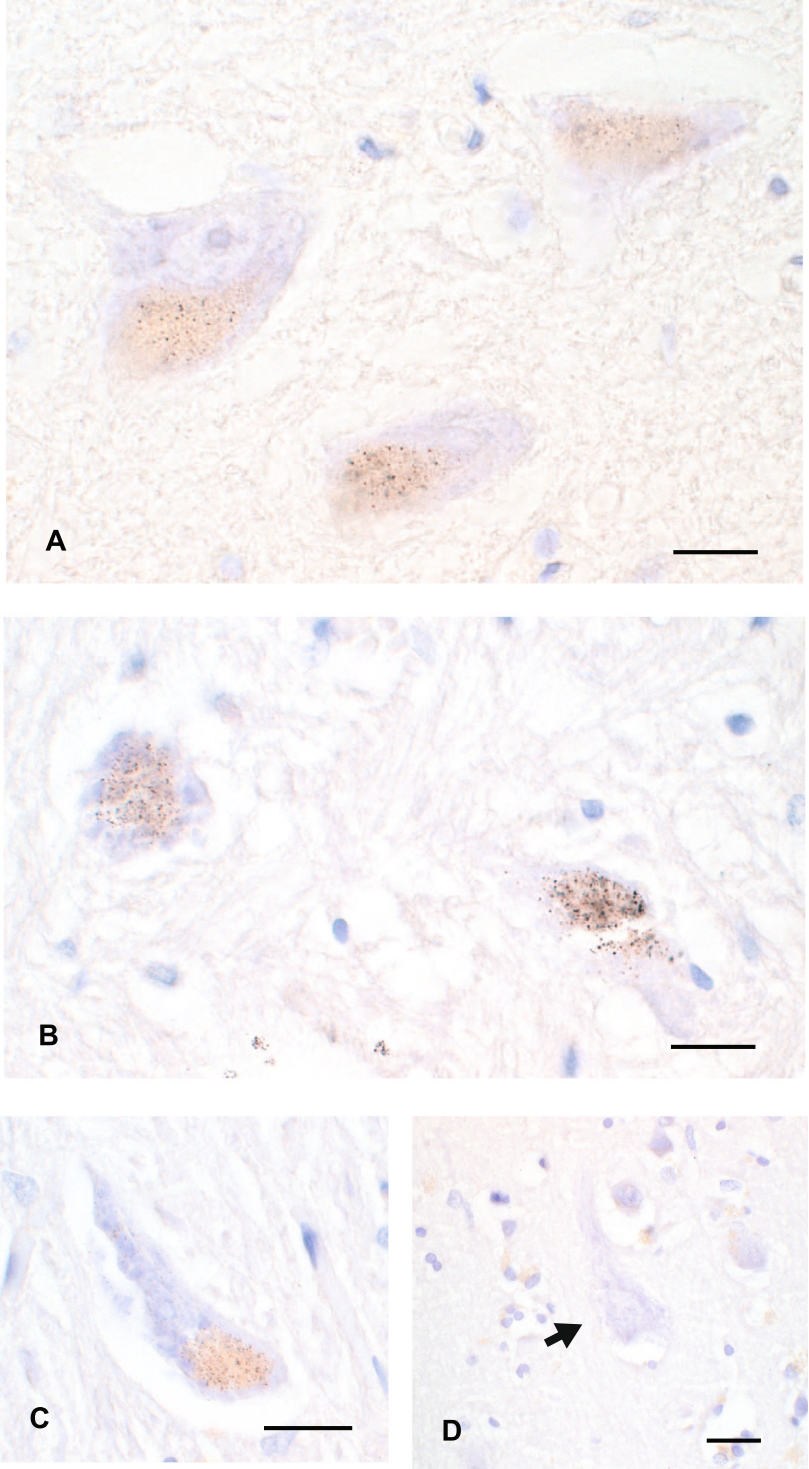
**Heavy metal staining in MND brain stem and cortical motor neurons. (A)** Light AMG staining is present within the lipofuscin of three hypoglossal motor neurons. Bar = 15 μm. **(B)** These two nucleus ambiguus neurons contain light AMG staining. Bar = 20 μm. **(C)** Light AMG staining is seen within the lipofuscin of a trochlear nucleus neuron. Bar = 25 μm. **(D)** A shrunken cortical motor neuron (Betz cell, arrow) contains no AMG grains. Bar = 15 μm. AMG and hematoxylin.

All 12 individuals with hypoglossal motor neuron AMG staining (10 out of 24 MND patients, 2 out of 4 controls) also had AMG-stained spinal motor neurons (Figure 5A). On the other hand, 6 individuals who had AMG-stained spinal motor neurons had no hypoglossal motor neuron staining. Spinal motor neurons therefore appeared to take up metal toxicants more avidly than hypoglossal motor neurons.

#### Heavy metals in nucleus ambiguus motor neurons

Nucleus ambiguus neurons stained positively for heavy metals in 8 out of 22 SMND patients where the nucleus could be identified, and in 1 out of 4 controls (Figure 5B). Heavy metal staining in the nucleus ambiguus followed that in spinal motor neurons, apart from one SALS patient who had positive nucleus ambiguus staining but negative staining in LC, spinal motor and hypoglossal motor neurons.

#### Heavy metals in extraocular muscle motor neurons

Heavy metal staining in extraocular motor neurons, either trochlear or oculomotor, was present in 6 (27%) of 22 MND patients (Figure 5C), and in 3 of 4 controls (2 of these having Parkinson’s disease).

#### Heavy metals in cortical motor neurons

No remaining cortical motor neurons contained AMG grains in any MND patient (Figure 5D). No glial AMG staining was present in the vicinity of these neurons.

### Heavy metals in other neurons

**Figure 6:**
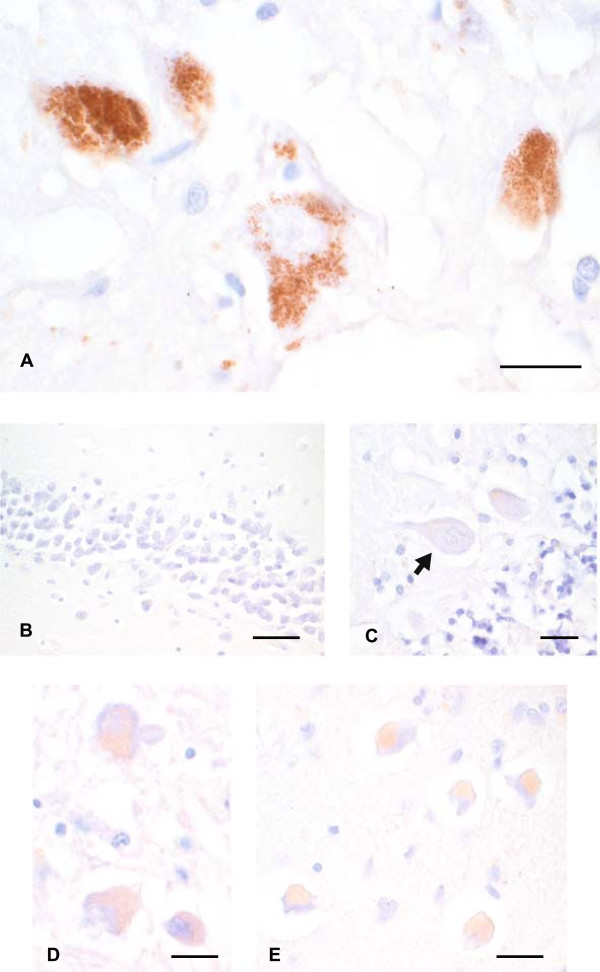
**Negative heavy metal staining in different CNS regions (all from the same MND patient in Figure**1**and Figure**5**B, C and D).** No heavy metal staining was present in: **(A)** Neuromelanin-containing neurons of the substantia nigra. Bar = 25 μm. **(B)** Neurons in the dentate gyrus of the hippocampus. Bar = 50 μm. **(C)** Purkinje cells (arrow) in the cerebellar cortex. Bar = 20 μm. **(D)** Lipofuscin-containing neurons in the cerebellar dentate nucleus. Bar = 25 μm. and **(E)** Lipofuscin-containing neurons in the inferior olivary nucleus. Bar = 30 μm. AMG and hematoxylin.

An occasional neuromelanin-containing neuron in the medulla oblongata contained heavy metals, in individuals in whom either AMG-stained LC or spinal motor neurons were also present. No heavy metal staining (not even a single grain) was seen in pigmented or non-pigmented neurons of the substantia nigra (Figure 6A) in any individual, including the 3 patients with Parkinson’s disease. No heavy metal staining was seen in any individual in neurons of the occipital cortex, hippocampus (Figure 6B), caudate-putamen, or cerebellum (Figure 6C). Neurons that contained abundant lipofuscin, such as those in the cerebellar dentate nucleus (Figure 6D) or the inferior olivary nucleus (Figure 6E), also contained no heavy metals. No heavy metals staining were seen in glial cells in any CNS region.

## Discussion

This study suggests that human LC neurons contain heavy metals quite commonly. These LC heavy metals are, however, more likely to be found in patients with MND than controls. In the majority of MND patients, heavy metals were detected in spinal motor neurons as well. The AMG silver amplification technique used detects the sulphides or selenides of mercury, silver, and bismuth. After silver was removed chemically from the tissues, AMG staining was still present, indicating that either mercury and bismuth was likely to be causing the staining. Human exposure to bismuth is rare, occurring mostly after consumption of bismuth-containing medications [12]. Therefore the metal seen on AMG in this study is most likely to be mercury, a known neurotoxicant with numerous natural and anthropogenic sources [13].

### The locus ceruleus is a potential pathway for toxicants to enter the brain

Experimental animal studies show that circulating metal toxicants enter motor neurons selectively, probably via retrograde axonal transport from the neuromuscular junction [14]. However, only recently has it been shown that a heavy metal, inorganic mercury, enters LC neurons selectively as well [2]. This entry into the LC probably occurs because the LC innervates the great bulk of CNS microvessels [15], and so is exposed to a large volume of circulating blood. In fact, a rough calculation indicates that, with the number of normal human LC neurons being 32,000 [16] and the total capillary length of the brain being 640 kilometres [17], each LC neuron on average innervates a 20 meter length of capillary [18]. The LC contains numerous neurotransmitters in addition to noradrenaline [19], and re-uptake of these neurotransmitters at capillary terminals could be harnessed by a number of toxicants to enter the LC via recycling mimicry [20].

### Age and heavy metals in the locus ceruleus

In our previous study, we found no heavy metals in infant motor neurons, and suggested that metals were likely to accumulate in motor neurons during aging [3]. In the present study, there was no correlation between increasing age and heavy metal content of the LC. This raises the possibility that toxicant exposure in these individuals was episodic, rather than continuous, in nature. Exposure may occur early in life, as evidenced by one 26 year-old man without neurological disease already having heavy metals within his LC. One possible reason for this early LC uptake of toxicant is that stressor-induced upregulation of the LC promotes bursts of toxicant intake into the nucleus [2].

### The amount of intracellular heavy metal varies widely between adjacent locus ceruleus neurons

A question that cannot be answered by this study was why only some LC neurons contained heavy metal staining, with densely-stained neurons usually found adjacent to neurons with no AMG staining. This phenomenon was also seen in a man who injected himself with a large dose of metallic mercury [2]. Topographic outputs of the rodent and primate LC to various brain regions have been described, but not at the level of individual LC neurons. Possibilities are that the toxicant-containing LC neurons have a particularly large output to the capillary bed and so are exposed to greater amounts of circulating toxicant. Another possibility is that specific stressors, e.g., those requiring the LC to activate the motor system, result in the uptake of toxicants into LC neurons that are activated to deal with those specific stressors.

### Could toxicants in the locus ceruleus trigger MND?

**Figure 7:**
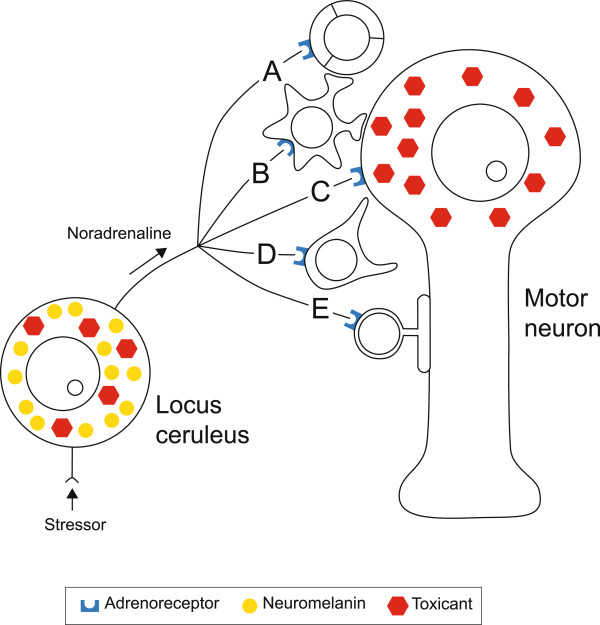
**Potential interactions between toxicant-containing LC and motor neurons (see text for details).** Toxicant uptake into the LC could reduce noradrenaline output, resulting in: **(A)** A dysfunctional blood-brain barrier, which becomes permeable to the same or other toxicants, or to inflammatory agents. **(B)** Decreased astrocytic function leading to increased synaptic glutamate. **(C)** Reduced trophic support to the neuron. **(D)** Microglial activation and inflammation. **(E)** Oligodendrocyte dysfunction causing impaired myelination.

We were not able to detect heavy metals in cortical motor neurons, despite these neurons being affected in ALS, and being shown to contain mercury after exposure to metallic mercury [2]. This could be because of a survivor effect, with the toxicant-containing cortical motor neurons dying early and leaving only toxicant-free motor neurons intact. Secondly, toxicants within the LC may reduce noradrenaline output to the cortical motor neurons and secondarily damage cortical motor neurons, without the need for a toxicant to be within cortical motor neurons. After being activated by a stressor, LC neurons supply noradrenaline directly to motor neurons, as well as to microvessels and glial cells, all of which have noradrenaline surface receptors (Figure 7).

Recent studies indicate that many CNS cells have noradrenaline receptors, so reduced noradrenaline output can cause a number of deleterious changes to neurons [19,21]. These include changes that are implicated in MND, such as blood–brain barrier permeability to toxicants or inflammatory agents [22], decreased astrocytic function leading to increased synaptic glutamate [23], reduced trophic support to the neuron [24], microglial activation and inflammation [25], and oligodendrocyte dysfunction causing impaired myelination [26]. Finally, it has been suggested that the LC could transfer toxicants from the circulation to cortical motor neurons [2], since the LC makes direct contact with both capillaries and motor neurons. A further discussion on how toxic damage to the LC could result in different neurological conditions has recently been published [18].

No technique currently exists to reliably measure the concentration of toxicants such as heavy metals within individual neurons, which raises the question of how to assess how much heavy metal is in LC neurons. The lack of obvious neuronal loss in the metal-containing LCs of our MND patients could lead to the presumption that the amount of heavy metal was insufficient to damage the neurons. However, it has previously been shown that mouse spinal motor neurons that contain a non-toxic dose of inorganic mercury suffer axonal shrinkage, without any loss of numbers of motor neuron cell bodies [9]. This indicates that a low dose of a heavy metal can cause neuronal damage without cell body loss, and by extrapolation suggests a loss of noradrenaline from LC terminals could occur without the loss of toxicant-containing LC cell bodies. Of interest, one of the few quantitative studies of the LC in MND has shown neuronal shrinkage of LC cell bodies without cell loss [27], though no unbiased quantitative studies of cell numbers in the LC of MND patients have been reported. Even a moderate level of LC neuronal damage could have a deleterious effect on spinal motor neurons, since the long LC axons extending down to the spinal cord would be particularly susceptible to toxic disturbances in their parent cell bodies [28].

AMG can detect only a few heavy metals, but environmental exposures usually involve different types of toxicants, often simultaneously. For example, cigarette smoke, a possible risk factor for MND [29], contains 4,800 identified compounds, including metal toxicants [30]. The heavy metals identified in this study may not therefore be a cause of neurotoxicity, but may merely be a marker that indicates how readily a number of toxicants are entering LC and motor neurons.

Although we have suggested here that heavy metals in the LC could play a part in the pathogenesis of MND, an alternative explanation for the presence of heavy metals in the LC is that metals enter the LC after the start of the disease. Stress-induced uptake of toxicants could take place after the onset of MND, because having a disease such as MND is likely to be highly stressful. The finding of heavy metals in the LC could therefore be a result of having the disease, rather than being a trigger for the disease.

### Heavy metal staining in extraocular muscle neurons

Extraocular muscles are affected late or not at all in MND [31]. Extraocular muscle neurons contained the least heavy metal staining of all the motor neurons in the present study, raising the possibility that one reason they are spared is because they take up smaller toxicant loads. This may be because their neuromuscular junctions are different to those of other muscles [32]. Curiously, 2 out of 3 Parkinson’s disease patients had heavy metals in their extraocular muscle neurons; whether this is related to the oculomotor problems associated with Parkinson’s disease [33] would require further study.

### Phenotypic variation in familial and sporadic MND: do toxicants play a part?

Heavy metal-containing neurons were found in this study in familial, and not only sporadic, MND. One possibility for this is that FMND-associated mutations increase the neuronal uptake of toxicants, possibly by altering the permeability of the blood–brain barrier as has been suggested for *SOD1*[34], *TARDBP* and *ANG* mutations [35]. Other FMND mutations could damage mechanisms that protect neurons from toxicants, as has been suggested for a number of MND genetic variants [36].

The presence of toxicants within LC and motor neurons could also explain the intra-familial variations in age of disease onset and phenotype that is seen in FMND, as well as the inter-patient variations commonly found in sporadic MND. For example, we found that the heavy metal content varied between hypoglossal and spinal motor neurons, a possible reason why bulbar symptoms can appear early, late or not at all in MND.

### Heavy metal staining in non-MND disease controls

With the recent finding of LC damage in multiple sclerosis [37] it is of interest that the LC of one multiple sclerosis patient contained heavy metals. The heavy metal staining within the LC of one individual with alcoholism accords with the finding by some workers that LC neurons are damaged by alcohol excess [38].

### Identification and quantification of heavy metals in neurons

Ideally, AMG should be followed up with some elemental method to confirm the identity of the heavy metal in the tissue. This has been achievable in humans exposed to large amounts mercury or bismuth, where enough of the metal was present in the tissues for quantitative methods to be of use [12]. However, AMG, being an amplification technique, is more sensitive to the presence of toxicant metals than other currently available techniques of metal detection (such as neutron activation analysis, proton-induced X-ray emission, atomic absorption spectrophotometry, and electron emission X-ray spectrophotometry), though the lower amount of heavy metal that can be detected with AMG has not been ascertained [10]. Unfortunately, microprobe elemental analyses performed on formalin-fixed paraffin sections are considered to be unreliable [39]. In our study, only a small proportion of the total number of cells in the pons, brain stem and spinal cord had AMG staining, which suggests only an *in situ* microprobe technique, carried out on frozen sections, would have a chance of detecting toxicants in the LC. Motor neuron loss in most MND cases was too severe to be able to identify toxicants using elemental analysis.

AMG has been used extensively to study the distribution of metal toxicants in experimental animals, used occasionally in humans with known heavy metal exposure [2,12,40], but used only rarely in humans with no known toxicant exposure [11]. Humans differ from most non-primate animals in having extensive neuromelanin pigmentation in catecholaminergic neurons, as well as having lipofuscin in many aging neurons, including motor neurons [41]. It is therefore important to ensure that the AMG staining seen in human neurons is related to heavy metal staining, and not to some unknown reaction within pigmented neurons. The fact that no heavy metal staining was seen in any neuromelanin-containing neurons of the substantia nigra, or in other neurons containing high levels of lipofuscin, indicates that these pigments themselves are unlikely to be the source of the AMG staining.

The heavy metals in this study localised preferentially to the neuromelanin of LC neurons, and to the lipofuscin of motor neurons, probably because these pigments can bind metals [41]. Neuromelanin in the substantia nigra of neurologically-normal individuals has an affinity for a number of physiologic and exogenous metal ions, including mercury [42]. It has been suggested that the neuromelanin of the substantia nigra could play different roles in Parkinson’s disease, firstly as a protectant by scavenging toxic substances such as exogenous metals and pesticides, and later as a destructive agent when cell death releases the neuromelanin [43]. Neuromelanin granules are probably not membrane-bound, and toxicants in unbound neuromelanin would be free to interact with cellular constituents [41].

Quantitation of the metal content of neuromelanin isolated from the substantia nigra of neurologically-normal individuals shows how neuromelanin can sequester toxic metals that arise from environmental exposure, with many-fold accumulations of mercury (1:96) and lead (1:1408) compared to surrounding tissue (no corresponding data are available for LC neuromelanin) [44]. Of note, however, was the absence of stainable heavy metals in any substantia nigra neurons in our study, despite the probability of age-related heavy metal sequestration by neuromelanin in these neurons. This suggests that the AMG staining we did see in the LC, especially in densely-stained neurons, represents a concentration of heavy metal that could be physiologically active. Indirect evidence of the potential toxicity of the level of AMG staining seen is also given by the findings from a man who injected himself with a large amount of metallic mercury [2], after which similar AMG staining of many LC neurons was seen, comparable in particular to the densely-stained neurons in the present study.

### Study limitations

Our study has a number of limitations: (1) The number of controls who had both LC and spinal cord tissue available was limited, since spinal cord tissue was usually removed at post mortem from controls only when clinically indicated, and LC-containing blocks were not available from all potential controls. (2) Only one (variable) level of the LC was available for examination, so we were unable to determine if differences in heavy metal content were present in rostral, middle and caudal parts of the LC; of note, cell loss in the LC in Alzheimer's and Parkinson's diseases has been found to vary along the length of the LC [45]. (3) We could not reliably detect the subceruleus, which in primates is thought to innervate spinal motor neurons [46]. (4) No histories of toxicant exposure were available, so we do not know if any individuals had exposure to higher than normal levels of mercury (e.g., living near an incinerator or a coal-burning power station) or had been taking bismuth-containing medications. (5) No psychological, psychiatric, or stressful life events histories were available, so we were unable to determine levels of stress before the onset of MND. This is of potential importance, since it has been suggested that stress, by activating the LC, could increase the uptake of toxicants into LC neurons [2]. (6) As noted above, we were unable to identify the type of heavy metal using paraffin sections, though this is likely to be inorganic mercury since human exposure to bismuth is limited.

### Future studies

The finding of potential disease-causing toxicants in a CNS region that is not morphologically damaged by MND has implications for further neurotoxicological investigations into this disease. Attempts to locate toxicants in MND tissues have generally been unsuccessful, probably because toxicant-containing motor neurons have largely disappeared by the time of death, and bulk analysis of tissue is unlikely to detect toxicants affecting a small percentage of cells. An intact LC, however, is compact and easily located, and would be an ideal candidate region for newer microanalysis methods that can detect a range of toxicants [47].

The heavy metals within LC neurons in the present study did not appear to cause structural damage to the neurons, and only minor changes in LC neuronal size have been reported in MND [27,48]. There are however a number of neurodegenerative and psychiatric disorders where structural damage to the LC with associated noradrenaline deficits have been described. Chief among these are Parkinson’s disease, Alzheimer’s disease, major depression and bipolar disorder [18,49,50]. In Alzheimer's disease it has been suggested that the LC is the first region of the brain to be involved by the disease, and at a very early age [51]. Environmental toxicants have been associated with all these disorders, and a search for toxicants within the LC of these patients could yield interesting results.

## Conclusion

In conclusion, we have shown that the uptake of heavy metals by the human LC is a fairly common occurrence, and is seen more in MND patients than controls. The combination of toxicant uptake by the LC and lower motor neurons could result in damage to cortical and lower motor neurons. Toxicant-induced LC damage with subsequent decreases of noradrenaline may explain the involvement in MND of multiple CNS regions and cell types. Further studies using newer methods of elemental analysis should be able to inform us as to whether a range of toxicants resides in the LC of MND patients, as well as in other diseases where the LC is damaged.

## Abbreviations

AMG: Autometallography; FMND: Familial motor neuron disease; high^%AMG^: Category of locus ceruleus with 4% or more of AMG-stained neurons; low^%AMG^: Category of locus ceruleus with fewer than 4% of AMG-stained neurons; LC: Locus ceruleus; MND: Motor neuron disease; SALS: Sporadic amyotrophic lateral sclerosis; SMND: Sporadic motor neuron disease; SPMA: Sporadic progressive muscular atrophy.

## Competing interests

The authors declare that they have no competing interests.

## Authors’ contributions

RP conceived the study, participated in its design, undertook its coordination and drafted the manuscript. SKJ participated in the study’s design and carried out the autometallography. Both authors read and approved the final manuscript.
